# Making the most of animal data – improving the prospect of success in pragmatic trials in the neurosciences

**DOI:** 10.1186/1745-6215-12-S1-A102

**Published:** 2011-12-13

**Authors:** Kieren J Egan, Emily S Sena, Hanna M Vesterinen, Malcolm R Macleod

**Affiliations:** 1 Department of Clinical Neurosciences, Centre for Clinical Brain Sciences, University of Edinburgh, Royal Infirmary of Edinburgh, UK

## Introduction

Developing disease modifying treatments for neurodegenerative disease and stroke has proved remarkably difficult. It may be that current structures in the pharmaceutical industry encourage early clinical trials designed on the basis of exciting but incomplete preclinical data in order to protect competitive advantage. An overly generous reading of animal data may lead to underpowered clinical trials testing treatments at inappropriate time points and at ineffective (but side-effect free) doses. Trials based on a systematic analysis of animal data may have a better prospect of success. Here, we report such an analysis of interventions tested in transgenic models of Alzheimer’s disease (AD).

## Materials and methods

(1) Electronic searching of three online databases to identify publications reporting the use of interventions in transgenic models of AD where outcome was reported as behavioural (probe phase of the Morris water maze (MWM)) or histological (changes in immunohistochemically stained plaque burden) end-points. (2) DerSimonian and Laird random effects meta-analysis and stratified meta-analysis.

## Results

We identified 428 publications testing 353 interventions across 55 different transgenic models of AD. Study quality was low; 16% of papers reported random allocation to group, 22% reported a blinded assessment of outcome and no publications reported a sample size calculation. Blinded assessment of outcome was associated with lower effect sizes for results from the probe phase of the MWM. Longer durations between treatment onset and outcome assessment for plaque burden, and younger ages at assessment of behavioural outcome were each associated with lower effect sizes.

## Conclusions

Both study quality and study design characteristics appear to impact observed effect sizes. Study quality was low, and this was associated with larger estimates of treatment effects when a behavioural outcome was measured. Improvement in behavioural outcome (which may be influenced by effects on underlying pathophysiology or by effects on performance in the face of a fixed deficit) was larger in older animals, but improvement in histological outcomes was smaller with longer intervals between treatment onset and outcome assessment.

These findings highlight the importance of a detailed and systematic analysis of animal data before embarking on clinical trial. Specifically, if data from animal studies are to be invoked in support of a trial protocol investigators should be able to demonstrate that such data are largely free from bias, and that the treatment has shown efficacy under the circumstances (for instance the stage of disease) in which it is proposed that it be tested.

